# Designing a Serious Game (Above Water) for Stigma Reduction Surrounding Mental Health: Semistructured Interview Study With Expert Participants

**DOI:** 10.2196/21376

**Published:** 2022-05-19

**Authors:** Rina R Wehbe, Colin Whaley, Yasaman Eskandari, Ally Suarez, Lennart E Nacke, Jessica Hammer, Edward Lank

**Affiliations:** 1 Human Computer Interaction for Social Good (HCI4GOOD) Faculty of Computer Science Dalhousie University Halifax, NS Canada; 2 Methods Group, Human Computer Interaction Lab Cheriton School of Computer Science, Faculty of Mathematics University of Waterloo Waterloo, ON Canada; 3 The Games Institute University of Waterloo Waterloo, ON Canada; 4 Michael G DeGroote School of Medicine McMaster University Hamilton, ON Canada; 5 School of Pharmacy University of Waterloo Waterloo, ON Canada; 6 Faculty of Science University of Waterloo Waterloo, ON Canada; 7 Faculty of Applied Health Sciences University of Waterloo Waterloo, ON Canada; 8 Human Computer Interaction (HCI) Games Group Stratford School of Interaction Design and Business Faculty of Arts, University of Waterloo Waterloo, ON Canada; 9 OH! Lab Human Computer Interaction Institute Carnegie Mellon University Pittsburgh, PA United States; 10 Équipe LOKI, Inria Lille-Nord Europe University of Lille Lille, Villeneuve d'Ascq France

**Keywords:** human–computer interaction, games for change, games for mental health, sensitive topics, game design, empirical analysis, expert participants

## Abstract

**Background:**

Although in many contexts unsuccessful games targeting learning, social interaction, or behavioral change have few downsides, when covering a sensitive domain such as mental health (MH), care must be taken to avoid harm and stigmatization of people who live with MH conditions. As a result, evaluation of the game to identify benefits and risks is crucial in understanding the game’s success; however, assessment of these apps is often compared with the *nongame* control condition, resulting in findings specifically regarding entertainment value and user preferences. Research exploring the design process, integrating field experts, and guidelines for designing a successful serious game for sensitive topics is limited.

**Objective:**

The aim of this study is to understand which elements of game design can guide a designer when designing a game for sensitive topics.

**Methods:**

To carefully probe the design space of serious games for MH, we present Above Water (AbW), a game targeting the reduction of stigma surrounding MH, now in its second iteration. The game, AbW, serves as a consistent research probe to solicit expert feedback. Experts were recruited from a range of topic domains related to MH and wellness, game design, and user experience.

**Results:**

By using this deployment as a research probe, this study demonstrates how to synthesize gained insights from multiple expert perspectives and create actionable guidelines for successful design of serious games targeting sensitive topics.

**Conclusions:**

Our work contributes to a better understanding of how to design specialized games to address sensitive topics. We present a set of guidelines for designing games for sensitive subjects, and for each guideline, we present an example of how to apply the finding to the sample game (AbW). Furthermore, we demonstrate the generalizability to other sensitive topics by providing an additional example of a game that could be designed with the presented guidelines.

## Introduction

### Background

Anyone discussing topics such as mental health (MH), poverty, socioeconomic status, homelessness, race, sexuality, identity as a person of color, and sexual health will likely agree that calling the conversation *difficult* is an understatement. Often, these topics may be considered extremely personal or *taboo* in our society; in other words, these are *sensitive* topics. Research on sensitive conversations explores the approaches, techniques, and outcomes of these situations and emphasizes the importance of having these discussions [1-3].

Above Water (AbW) is used as a probe to effectively elicit insights from experts. We then demonstrate how to apply these insights to the game, AbW, to exemplify how to design games that target sensitive topics. Contributions of this work demonstrates how to synthesize insights from multiple perspectives and create actionable items. We probed experts with different perspectives using a constant probe stimulus (AbW), which allowed us to capture the different perspectives from a range of experts and explore how these lessons may be generalized to other games targeting sensitive topics.

In this paper, we focus on the sensitive topic of MH. In our current sociocultural context, MH is often a difficult conversation. Researchers have demonstrated that parents need to have conversations early, to attempt to mitigate negative outcomes [4]. Untreated MH conditions are damaging to the individual and have cumulative effects on families, communities, business, and society. Treatment and support have significant tangible and intangible benefit to society. Considering only economic factors in the United States, the National Alliance on Mental Health reports US $193.2 billion in lost earnings per year owing to poor or unavailable treatment options. Moreover, this total does not speak to the additional nonmonetary benefits of increased mental wellness across society [5]. Although difficult to discuss, MH is an undermined aspect of overall health that is often overlooked in our culture, society, and sometimes even in the health care system itself.

Treatment for individuals is available; however, an individual may hesitate to seek treatment because of the stigma or the untrue, falsely held beliefs regarding MH [6,7]. It can be argued that the stigma and the associated feelings of shame, isolation, and misunderstandings perpetuated our culture and media. It can be argued that stigma causes more harm than the condition itself [8]. Stigma results in an avoidance of treatment and education about MH issues, thereby limiting an individual’s willingness to get help for themselves [9] or offer effective help to others [10].

Research has shown that it is possible to reduce stigma through the introduction of resources, new information, or meeting with individuals [11]. In other words, increasing MH literacy (MHL) is inversely related to stigma. Increased MHL or knowledge of MH also improves one’s ability to offer help [10]. In this paper, we explore how to increase MHL and reduce stigma in the context of a hybrid card and digital game. We leverage games and playful design to facilitate education and conversation.

The study by Juul [12] explains that games allow for the formation of a *magic circle*, a safe, playful space that encourages exploration. Within the magic circle, players can safely make mistakes and act in ways that are not in accordance with their everyday persona, thereby allowing the game to become an ideal environment for learning. Research on games and games for change demonstrates that learning through games can change behavior [13]. However, owing to the sensitive nature of MH and surrounding stigma, designing games for MH presents a particular challenge not seen with other health topics (eg, exergames) [14,15].

To understand what game mechanics and experiences would be most helpful, we invited experts in both MH and game design (GD) to participate in semistructured interviews aimed at eliciting information through the dismantling of a game designed to battle MH stigma. In this study, we use the game, AbW, as a research probe [16,17].

Through the dissection of the game, AbW, we see emergent themes of comfort, learning system, and technical design that led us to the presentation and discussion of successful mechanics for the design of sensitive topics. In this paper, we present design guidelines and demonstrate how to implement our guidelines using AbW as a case study. To further communicate the application of our guidelines and the applicability of these guidelines beyond MH, we also include a second example of a game that could be designed to tackle another sensitive topic.

### Literature Review

MH is an important part of overall health. According to Mental Health America 2018 [18], 18% of adults or 43.3 million Americans are living with diagnosable MH concerns. Similarly, the Mental Health Commission of Canada has identified that 20% of Canadians will also experience an MH concern in their lifetime [19]. MH concerns are not limited to adults; Mental Health America 2018 reported that 11.93% of youth (aged 12-17 years) have had at least one episode of major depression [18]. These statistics are alarming given that untreated MH concerns can lead to further health and social challenges, including substance abuse disorder, loss of employment, reduced social functioning, thoughts of self-harm, and death by suicide. Furthermore, untreated MH concerns can also lead to community-level challenges, potentially resulting in greater rates of incarceration and increased strain on national health care systems [18]. In addition, companies may experience a loss of productivity or high turnover rate of employees owing to untreated MH concerns, resulting in higher personnel recruitment and training costs.

The current understanding of mental illness reflects the complex biological, psychological, and sociological factors that impact MH, in what is termed as the biopsychosocial model. Biological explanations include neurochemical imbalances, impairments of neural networks, and inherited genetic predispositions [20]. In addition, environmental factors interact with genetic aspects that ultimately result in complex networks of factors potentially predisposing an individual to mental illness [20].

A broad range of environmental factors are understood to potentially contribute to MH challenges. Some factors can be substance-mediated, such as substance abuse, whereas others are rooted in one’s social environment and include adverse childhood events, sexual abuse, or trauma [21]. Literature on MH clearly shows that that both factors, internal and external to an individual, contribute to their MH [20,22].

Individuals living with an MH illness are less likely to receive treatment than those living with a physical health illness. It is estimated that only 20% of individuals with an MH concern or diagnosed condition sought medical treatment, and that only one-fifth of Canadian children who need MH care receive it [19,23]. In addition, approximately 60% of Canadians do not receive timely diagnosis and treatment (ie, <1 year from symptom onset) [24]. MH conditions represent serious challenges to those who live with them and our society at large.

The median time of diagnosis from the onset of symptoms is 4.4 years for depression and 6.2 years for anxiety disorders [24]. The time gap is even higher for illnesses such as schizophrenia and bipolar disorder [24].

Considering that MH illnesses are treatable and manageable, the previously mentioned statistics are disheartening. The effects of stigma or untrue beliefs perpetuated about a particular condition provide a possible explanation for this phenomenon.

### Effects of Stigma on MH Issues

Stigma around MH has been shown to result in individuals not seeking support or treatment, even when they are readily available [6,7,9,25]. For example, youth may struggle to seek help from a parent, teacher, or caregiver concerning thoughts of self-harm and suicide. After seeking help, stigma may cause individuals to feel that their own problems with anxiety are very embarrassing to share with a support group, thereby continuing the perpetuation of MH stigma. Therefore, reducing stigma can lead people to seek treatment or get help from their community.

Stigma is perpetuated in society through multiple methods, not limited to media, including television and video games [8]. The root causes of MH stigma are complex and actively perpetuated in western media [8]. Even promoting an understanding of the causes of mental illness can lead to stigma and have adverse, unexpected effects as people may view MH illness as something out of the control of people with MH illnesses, promoting the stereotype of unpredictability [26].

Although a better understanding of the biology alleviates negative feelings of blame for those experiencing the mental illness, resulting feelings of pessimism for the prognosis have also been noted [26]. Improving MHL is a key factor for reducing stigma; however, it must be done comprehensively to ensure efforts to reduce stigma are successful [23]. Studies have shown that this is possible through the introduction of resources, new information, or meeting with individuals [11]. Therefore, a discussion group with new information and connecting individuals with different lived experiences with mental illness can potentially reduce stigma. Sensitive topics, such as MH, require a safe environment for discussion. Consequently, the ability to access information and ask questions while preserving privacy of individuals is imperative for success.

If intervention is delayed, for example owing to stigma, one disorder is likely to progress to multiple comorbid disorders that are more difficult to treat, with higher chances of recurrence [27]. In particular, anxiety disorders tend to have a longer delay, from years to decades, owing to early onset and lower perceived need for treatment [27]. The solution is changing peoples’ beliefs about MH. Therefore, we need to explore platforms that provide a safe place to allow for learning, increase motivation, and change behavior.

### Platforms for Battling MH Stigma

Social media has the potential to be used as an MH stigma–fighting platform. The most notable form of this strategy has been the sharing of narratives, especially testimonials from those experiencing mental illnesses [28]. Johnson et al [28] showed that presenting content in the form of a narrative combined with homophily in social media is more effective in education about MH than simply stating facts and statistics. Social media interactions foster a peer-to-peer support system that can be helpful in allowing individuals to share similar experiences and can even be beneficial for users who merely view the content anonymously [29]. However, information regarding MH illnesses tends to contain more inaccuracies and stereotypes than those regarding physical illnesses [30].

Direct MHL education campaigns have the potential to provide accurate information and have been shown to improve attitudes toward MH [30]. However, the effects were not lasting and did not improve the confidence of participants in helping and supporting those experiencing MH issues [30]. Moreover, the emphasis of the biological causes of mental illnesses seemed to encourage a helpless attitude and fear of unpredictability and dangerousness [30].

Campaigns and programs that have been successful rely on multiple platforms and approaches. For instance, Time to Change, an extensive outreach program to improve public attitude toward MH issues and reduce stigma improved the attitudes of 5.4 million people in the United Kingdom since 2008 [31,32]. Their approach involved national social marketing and grassroots-level programs in communities involving social contact with those affected by MH-related stigma [31]. Beyondblue is another example of an extensive national initiative founded in Australia with the aim to improve MHL [31]. One of their notable approaches was providing a platform for well-known actors to share their experiences with MH illnesses [31].

Methods of fighting stigma involving both contact and education may be the most successful [30]. Serious games involving discussion of MH topics combine the benefits of both forms of approaching MH stigma. Games can provide players with a safe environment [12] and change their attitudes and behaviors [13]. They can also provide players with a sense of belonging or community, which may allow for the discussion of more sensitive topics [33,34].

### The Use of Games Related to MH

Playful apps, including games, gamified apps, and simulations, have been used adjunct to therapy, often by gamifying a well-known method of clinical treatment such as cognitive behavioral therapy. The game SuperBetter [35] uses a point system and principles of cognitive behavioral therapy to reduce depressive symptoms [35]. Similarly, virtual reality (VR) has gained some traction for exposure therapy of phobias. Although playful, VR apps are not necessarily considered to be games owing to the missing cooperative or competitive aspect [36]. The application of games in therapy has gained support because games are known to be a vehicle for motivating behavior owing to the complex and responsive rewards systems built into their design [37-39].

Learning complex ideas is part of many game systems. For example, the ability to catch a Pokémon in a game is an abstraction of a larger probability equation. Therefore, games have often been leveraged for learning [40]. There is an abundance of support for educational games, serious games, and gamification [36,41,42]; these games and apps aim to computerize aspects of existing evidence-based therapies. More research needs to be done on their contribution to the effectiveness of therapy; however, they can be potentially useful for learning about MH.

Another category of games targeting the lay person or general public aim to improve MHL. For example, Stigma-Stop is a nonimmersive VR game in which each participant plays the role of a person who is facing adversity from a mental illness [43]. Information about the illness and symptoms is presented to the player, who receives feedback based on their decisions [43]. The game succeeded in helping players gain a better understanding of each of the conditions and is demonstrated to effectively reduce stigma [43].

Researchers have shown that when players enter the game world, they suspend their disbelief to become more open to concepts [12]. As a result, researchers have demonstrated that games can provide a chance for reevaluation of ideas [44]. Therefore, games can provide an opportunity to reevaluate beliefs held as inherently true regarding MH. Stigma-Stop [43] was found to be successful in debunking common misconceptions about certain MH illnesses; however, its long-term effects are yet to be evaluated to confirm a long-term reduction in stigma [43].

Cangas et al [43] suggest that contact with those experiencing a mental illness may be the missing element. Researchers tested a serious game to provide training for responding to MH concerns in the workplace [45]. Hanisch et al [45] reported that management staff who played the game had improved knowledge about MH. In both games tested by researchers, accurate information was provided, and the players could interact with other characters experiencing a mental illness. Moreover, testimonials of real people experiencing mental illness were provided as a supplemental video. Therefore, both the education and contact strategy were used to reduce stigma and improve knowledge of MH issues.

In addition, in the context of games, the actions of players including failures are relatively inconsequential. Unlike other commonly encountered scenarios, it is acceptable to fail or not know the correct answer in a game [46,47].

Overall, games and playful apps that provide training were proven effective [35,43,45,48]; however, these apps are not always accessible to the public. Moreover, it is possible that these games are not appealing to the public owing to the formality surrounding sensitive topics, which again contributes to perpetuating stigma by discouraging comfortable discussion.

### The Potential of Serious Discussion Games in Mitigating MH Stigma

Although games have been used alongside therapy or as a part of formal training to improve MHL, there is an unmet need for games that facilitate discussion of this serious topic aimed at the general public. Serious games for MH are continuing to expand in the field as more game designers are beginning to explore the use of gaming strategies that can increase the positive outcomes for MH issues [49]. However, most of these games are either not publicly available (ie, only available to registered health professionals [HPs]) or not clinically tested [49]. In response, this study aims to explore GD that can provide a safe environment for thoughtful discussion of MH that has the potential to reduce stigma.

More traditional board games are also being used to facilitate these discussions. For instance, the Learning Life Game [50] is a simple, noncompetitive board game, which randomizes questions for discussion. The goal of the game is to help players learn about themselves and others. The game hopes to teach problem-solving strategies by leveraging role-playing game (RPG) strategies, imagined situations, and hypothetical questions [50]. The purpose of this game is to help adolescent therapy groups better convey their feelings and communicate. Answering the questions in a group was found to elicit cooperative behavior from other group members, particularly when challenging questions or vocabulary were encountered [50]. Overall, this game was a helpful tool in helping adolescents develop verbal skills and provided structure for group therapy sessions [50]. However, as mentioned before, games designed for a formal therapy setting are not necessarily suitable or appealing to the public for whom the reduction of stigma is a more immediate issue.

If serious topics are to be discussed in an unsupervised and informal setting, game mechanics must work to provide a safe space to improve the quality of the discussion. Privacy is a crucial element that can be afforded through the mechanics of gameplay such as anonymity, randomization, or hidden information. For example, anonymity is a contributing factor to the execution of cheating behaviors; therefore, GD can choose to include anonymity as a mechanism and leverage it to direct the atmosphere of the game space [51].

GD has shown mixed results for anonymity as a mechanism to shape the play space. Cheating behaviors and competitive actions can be encouraged through the use of deception or anonymity [51]. In contrast, the permissive environment encouraged by anonymity can be used positively. For example, AbW leverages anonymity positively to foster communication about MH and to reduce fears of experiencing stigma while playing the game. Similarly, other studies have also indicated that anonymity can be used to create more welcoming environments. A study [52] on a web-based focus group, which discussed dating and intimacy for survivors of childhood cancer, highlights key design elements that can aid the discussion of serious or sensitive topics. It was found that participants preferred the anonymity that the chat provided. They also preferred groups with >2 people and a moderator to stimulate the conversation [52]. AbW is a novel game that combines these design features to construct the ideal environment for the discussion of stigma surrounding MH.

Finally, games can be used as vehicles for storytelling, which has been found to increase empathy [53]. Playing games cooperatively can also increase feelings of empathy [54,55] and reduce feelings of antifriendship or pleasure from the misery of others [56]. Emotions can be leveraged in the design of educational games [57], which AbW plans to do through the emotional journey that the players experience. Although simulated, real-emotion experiences can be felt through games and stories [21,53,58].

GD can use the created empathy to design the mood and atmosphere in their games. For example, consider *Brothers: A Tale of Two Sons* (Starbreeze Studios) [16], which uses the relationship between the 2 main characters to perpetuate the story and elicit empathy from its players. Following this strategy, AbW attempts to use personal connection and empathy as part of the GD. AbW attempts to bring together members of a group to share stories and experiences that would otherwise not be discussed in everyday conversations, in the hope of effectively reducing stigma surrounding MH challenges.

### Design

The game, AbW [17,59], was designed to help educate individuals about anxiety, specifically generalized anxiety disorder (GAD) and panic disorder (PD). Upon further iteration and in-house testing, the concept of the game was widened to include general information about MH. The current version of AbW intends to educate individuals on general MH factors within a wider breadth and focuses on anxiety in depth.

In summary, AbW uses both physical cards and an integrated digital component in the form of a website, which is intended to be used similar to a phone app. The game was designed to be an educational tool and not a treatment in and of itself. Potential use cases include support groups, caregiver–child dyadic pairs, and MH training.

AbW is designed to be an experience in which participants experience a range of emotions; for example, anticipation of getting the right card, relief for being able to ask desired questions, joy when discussing victories related in their own or their bespoke character’s journeys, and silliness when playing a minigame. Although the game is not designed to be inherently stressful, participants may converse in depth about situations that are stressful and choose to disclose personal information. The variance of emotional experience will depend on the group, setting, and individuals’ decisions to disclose information. Owing to the variability in conversation, the game has inherent replayability, because it is nearly impossible to replicate a human interaction as no 2 conversations are the same.

### Design History

The design of AbW has been detailed in previous work [16,60]. AbW began as a seminar project focused on exploring the designing of games for health. The GD was presented at 2 international conferences in the form of a student design competition submission [59] and an interactive demonstration [16].

Upon moving to evaluate the efficacy of the game as an educational game and an intervention for MH stigma, there were concerns about the safety of presenting information about MH to naïve participants owing to the sensitive nature of MH conversations. Researchers worked closely with the office of research ethics to determine the safest approach to evaluate the game. To ensure the safety of players, it was decided that an evaluation with expert participants should precede the evaluation of the game with a large group of naïve player-participants.

### Digital and Physical Card Game

**Figure 1:**
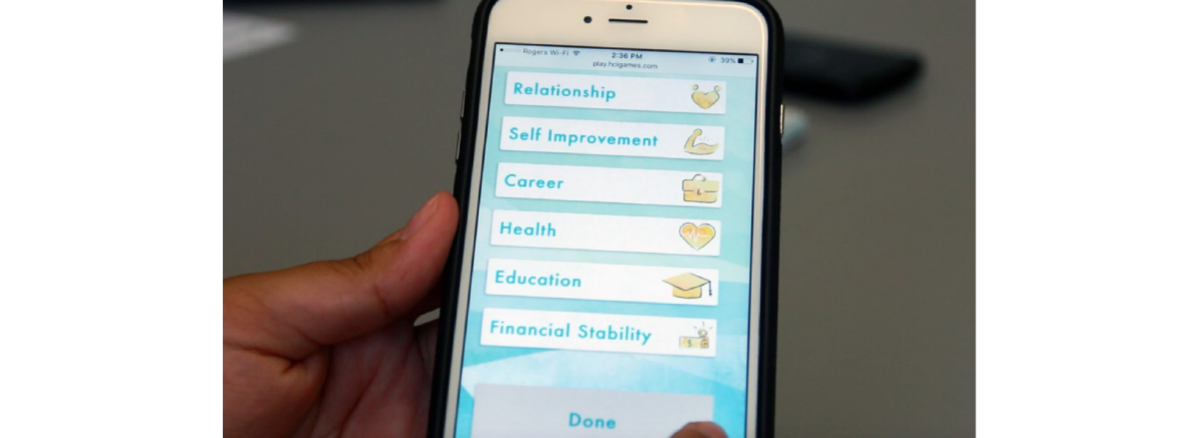
An image of the app, which comprises part of the gameplay space [17,59].

**Figure 2:**
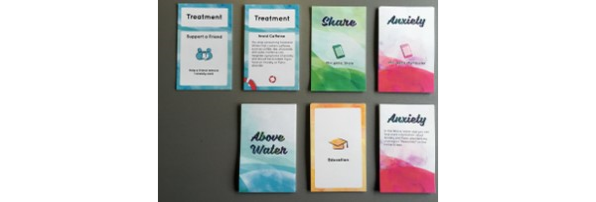
The card types in the game [17,59].

The game is played with a deck of cards and a personal computing device (ie, mobile phone or tablet; Figures 1 and 2 [17,59]). Most gameplay is conducted through physical game cards, and the game is moderated by the app.

The goal of the game is to collect the player’s chosen *life goal cards*. Among other functionalities, the digital component serves to track the *life goal* the players have selected.

**Figure 3:**
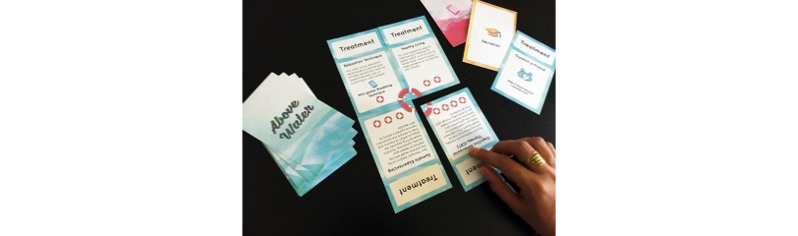
The treatment cards come together to symbolize the complexity of treatment plans, which usually comprise multiple interventions or habit-changing efforts [17,59].

Added challenge stems from the appearance of *anxiety cards*, randomly drawn from the deck. Management of *anxiety cards* is made possible with *treatment cards*, which convey information and remove the negatively-scored *anxiety* cards the player may accumulate as the group traverses the card deck (Figure 3 [17,59]). In-game effectiveness of *treatment cards* is rated based on the amount of help that each treatment requires from an MH professional. The more involved an MH professional is in the execution of the treatment, the more anxiety cards the treatment card can counteract. For example, a healthy eating card would only counteract 1 anxiety card, whereas the card representing a psychiatrist’s prescription would counteract significantly more.

**Figure 4:**
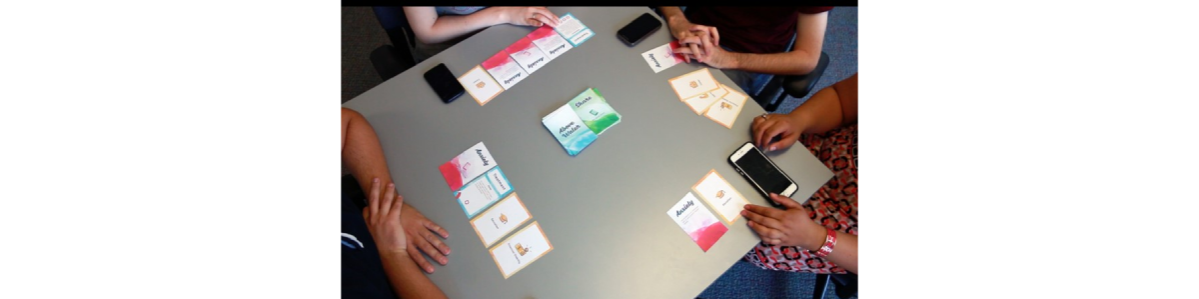
A picture of the game in play using the bring-your-own-device policy. Note that the game is played open-handed [17,59].

The game is played open-face, with accumulated *life goal* and *anxiety* cards displayed openly (face-up) on the table (Figure 4 [17,59]). The collected cards in front of each player denote the player’s current progress.

Finally, the last category of cards is the *share card* which asks users to share experiences, tips, or ask questions. The game facilitates dialogue though *share cards*, which are meant to stimulate discussion, self-reflection, and discourse with other players. The *share card* activity always points to the digital app.

The app serves to augment the card game. During the gameplay, players may draw a card with a phone-icon; this game event instructs players to use the mobile app. Treatment cards, share cards, and anxiety cards point to minigames in the digital component such as myth-busting, deep breathing, and yoga exercises.

Activities associated with these cards use the players’ phones for facilitation. These include engaging players in minigames to allow them to try some of the basic at-home methods of managing anxiety, such as guiding players through yoga poses and deep breathing exercises. The share cards ask players to type a response to a prompt in their phone, which are randomized and shared anonymously with other players.

By augmenting a traditional in-person card-based game with phone-based features, AbW seeks to provide players a way to fully engage in the game with other players to express feelings and talk comfortably about sensitive topics, while maintaining anonymity and privacy.

### Designing for Space and Privacy

The game features a distributed platform design, which spans the mobile device and a tabletop card game. We took a novel approach to the tabletop game paradigm by orienting and guiding the card game using a mobile app. We follow a bring-your-own-device (BYOD) protocol (Figure 4 [16,59]). The BYOD protocol allows players to have control over their own privacy. Participants can choose to access the web portal through the browser of their choice with a device calibrated to their preferred settings; for example, the participant can choose to use a privacy-aware browser or their browser’s *private browsing* mode.

The game is designed to foster discussion and protect anonymity of the users, while allowing for a shared group learning experience. The game leverages anonymity [51] through augmentation of the card game through a mobile app. The app allows users to input answers, and it randomly selects 1 answer to be presented for group discussion. The app balances anonymity through face-to-face group discussion. Both these design decisions, anonymity and in-person interaction, have demonstrable effects on the game’s atmosphere and were selected to reduce stigma associated with diagnoses of MH illnesses or MH concerns [11,61]. For a full description of the game mechanics, please refer to previous work [16,17].

### Accessing Trusted Resources

The GD is the contribution of the project, the content itself is not. The content is sourced from credible trusted resources such as medical organizations, government organizations, and outreach programs. Information presented in the game is designed to spark curiosity and encourage players to read about MH and pursue their own active education after the game ends. With the BYOD paradigm, players leave the game with the links to resources on their phone and can access the website again anytime.

## Methods

### Overview

In this study, we asked MH professionals and game designers to provide feedback on the game, AbW, through semistructured interviews.

The expert evaluation of our game allows the researchers to ensure that our intervention, AbW, is safe and has a likelihood of being effective before proceeding to conduct a playtest with a group of nonexpert participants.

AbW was used as a probe to effectively elicit insights from experts. We then demonstrate how to apply these insights to the game, AbW, to exemplify how to design games that target sensitive topics. Contributions of this work demonstrate how to synthesize insights from multiple perspectives and create actionable items. We probed experts with different perspectives using a constant probe stimulus (AbW), which allowed us to capture the different perspectives from a range of experts and explore how these lessons may be generalized to other games targeting sensitive topics.

### Participants

In total, 14 participants were interviewed for the study, 7 (50%) HPs and 7 (50%) game designers, between April 2018 and November 2019. Demographic information of the participants is shown in Table 1. We sought to speak with individuals with a variety of roles in MH care provision and in the games industry. Within the scope of MH professionals, we included social workers, counselors, and community leaders, who contribute to the biopsychosocial definition of health in the community; for example, MH3 is a hospital chaplain who services patients, families, and caregivers to support coping and provide counseling. In addition, expert participants from game, user experience (UX), and related disciplines were recruited internationally (North America and Australia).

Expert participants in MH and wellness were recruited by contacting health institutions and practices in the local community. Further calls for participation were sent to HPs nationally via professional network organizations, networking, message boards, and listservs. Recommendations from participants were also followed.

**Table 1:** Participants’ occupation.

Demographic type and ID			Demographic information
**Mental health professionals**			
	MH1	Clinical and counseling psychologist	
	MH2	Registered practical nurse	
	MH3	Chaplain (hospital)	
	MH4	School guidance counselor	
	MH5	Psychology professor	
	MH6	Clinical psychology lecturer	
	MH7	Occupational therapist	
**Game designers**			
	GD1	Game designer and experience designer	
	GD2	Game designer	
	GD3	Game designer	
	GD4	Experience designer	
	GD5	Game producer	
	GD6	Game designer and programmer	
	GD7	Senior user experience professional	

### Interview Protocol

Interviews were made accessible by allowing participants to choose how and where the interview was conducted, as web-based or in person, and at a time of their choosing. Consent was obtained before participating.

Participants were sent a digital copy of the game and a link to the game website to review before the interview. During in-person interviews, a physical copy of the card deck was also presented. Participants were informed that the game was still in the prototype phase, so that participants would feel comfortable to be critical of the game.

Interview questions focused on the benefits and risks of playing the game socially, in a clinical or professional setting; the feasibility of the game mechanics, especially the digital components; and the ability of the game to achieve its goal of reducing stigma. Expert participants were also asked to convey their opinions on the risks and benefits of AbW and games for change as interventions.

The interviews were semistructured. Prompts were included for the research team to pursue based on their own judgment. A copy of the provided prompts is included in Multimedia Appendix 1. Typical to semistructured interviews, information arising from past interviews provided direction for continued discussion. The interview was designed to take approximately 1 hour; however, in clinical and business settings, this time was often shortened. When interviews were shortened, we concentrated on larger themes (Multimedia Appendix 2).

### Interview Analysis

The interview protocol itself was semistructured, with prompts for continued discussion. Interviews were audio recorded and then transcribed. Interviews were coded using NVivo (version 12.2.0; QSR International) for macOS using thematic coding in a grounded theory methodology [62].

As data were collected, the researchers began to build theories and look for conversion within the emerging results, beginning with a line-by-line assessment for our initial 2 participants, which grounded the further thematic analysis with subsequent participants. Coding conducted on a line-by-line basis abstracted key themes from participant responses. Relevant codes were then organized into a mind map, allowing codes to be organized into emergent themes for inductive analysis. A positivist approach was taken during this analysis, as the data analyzer believed that evaluating participants’ thoughts about the game in an objective manner would result in conclusions about the game that would increase the game’s utility to stakeholders, including clinicians [63]. That is, viewing participant responses as impartially discussing the game allowed more generalizations to be made about it.

Upon completion of the data collection phase and transcription, thematic analysis based on the initial grounded theory results was used to understand larger themes. Three members of the research team (RRW, CW, and YE) reviewed the transcripts independently and decided the potential codes individually. Then, they merged their codebooks through discussion and reached consensus to form a preliminary codebook. These preliminary codes were then organized into 12 major codes inductively.

Then, to remove possible biases, a full code analysis was performed by YE and naïve coder AS, who coded all the interviews using NVivo (version 12; QSR International) for macOS, paragraph by paragraph using the major codes. The overall unweighted agreement among coders was *κ* of 0.67, indicating moderate agreement among coders.

Following the completion of this iteration of coding, RRW and CW rejoined the discussion, and then, all the 4 researchers discussed the results of the coding while providing their respective takeaways for each of the 12 major codes. Following the conclusion of this protocol, all the authors were invited to review the findings. The complete code reference is shown in Table 2.

**Table 2:** Qualitative coding reference table.

Theme and major code		Description of major code	Clinical or medical	GD^a^ or UX^b^	Minor codes
**Comfort**					
	Security and privacy	Owing to sensitivity of the data, precautions on how it is stored and accessed and how participants feel about sharing their information (Do they feel safe and trusting of the system and other players?)	58	54	Anonymity, privacy, risks, safety and vulnerability, sensitive information, checkpoints, and sharing
	Social dimensions	The social environment surrounding the game, including how participants feel (awkwardness and tension), talk to each other, room atmosphere, and the resulting consequences (ie, quality of conversation, fear of being incorrect, or challenging or correcting others).	72	49	Social environment, wrongness, conversation facilitation, and awkwardness
	Facilitator	Facilitator role or game leader role as it is needed or as the game is designed.	21	40	Authority, facilitator, and facilitator skill
	Clinical value or outcomes	The outcome of the game as it applies to players leaving the game (What did they learn? Did it affect their thoughts or behaviors?)	83	92	Game outcome and clinical effects
**People**					
	Community	The ability of the game to create a connected environment within a community. In this category, we refer to the community as a group with a clinical facilitator.	41	18	Clinical and community value of game
	Target	Who is the target population (age and familiarity with content) and what are their relation to each other? How does that affect the design of the game?	66	40	Population and age
	Replay or customization	Features adding to replay value or customization of the game.	11	24	Customization for player, clinician, or facilitator and replay value of game
	Content	The content of the game, specifically the density, depth, and breadth of the information.	90	87	Content of resources, game content, data dense, and data sparse
	Learning system	How learning is approached, handled, and reinforced. In this category we are specific to the learner (not the facilitator role).	60	42	Learning, scaffolding, pedagogy, question structure, presentation of content, and reinforcing
**Design**					
	GD	How the game is designed and the game’s attitude (ie, fun or cooperative).	103	163	Pacing, simplicity, fictional person and narrative, cooperative vs competitive goals, impact chain, strategy, analogy, and random
	Technical design	The design of the app itself (void of all learning and game elements). Here we refer to the technical elements of the interface (navigation, buttons, etc).	33	43	Mechanic of typing, keyboard accessibility, and input methods; autonomy; navigation of app; coordination; syncing; and hard to reference
	Onboarding	Onboarding procedures and clarity of instructions for both the facilitator and the learner.	22	28	Onboarding and instruction clarity

^a^GD: game design.

^b^UX: user experience.

### Ethics

This study received ethics approval from the University of Waterloo, Canada, registered under office of research ethics (number: 32195). The data set for this study is not available for access.

## Results

### Overview

The iterative process that resulted in the final codebook can be represented in 3 stages. In the first stage, the resultant codes of individual exploration of the data were compiled. After additional reflection and exploration of the data set, the authors proceeded to combine the codes into larger themes. Then, the themes resulting from this stage were used to code the data set. Next, line-by-line coding of the interviews with both medical and UX experts and the findings from each domain were discussed in detail. Finally, an overall look at the data set revealed the presented themes. In this stage, the authors discussed the resultant categories and developed 3 main theories. Each theory is described in detail in this section. Quotes are presented for each theme to illustrate the findings from the data.

### Comfort

#### Overview

Owing to the sensitive nature of the discussion topic, the research team worked closely with the office of research ethics to ensure that the content presented in the game was not triggering. Here, we use *triggering* to describe the negative effects that can occur with the presentation of a stimulus to a venerable party. As a result of discussions with the ethics board, it emerged that we would need to probe our expert participants for their assessment of safety and possible risks of AbW.

The first emergent theme from the data points to the importance of participant comfort. Upon analysis of the results, we see the emergent theme of participant comfort, which goes beyond our initial research question pertaining solely to the presentation of triggering information. As it became clear that our probe was very narrow, we began to collect data on factors that contribute to a concussive environment for discussion. The results indicated that comfort goes beyond the content provided on the cards. From the data, we see a clear need for an overall consideration of security; privacy of data; social environment, clinical and community values; and a facilitator.

#### Am I Comfortable With the System?

Does the player trust the game? We know from the literature that privacy and security on the web is a challenging topic for the nonspecialized, lay user to understand [64]. Therefore, part of the work in designing a game that connects to the internet is to ensure that users feel that data entered into the app will not be used maliciously. Designed with this in mind, the game does not store information or participant data. In addition, the game was designed to obtain user input and randomly select an answer without identifying the user themselves:

The anonymous component of it is very helpful, because then a group of people can get through a game session and not necessarily put them in a position where they’re - they’re vulnerable about something that might be difficult to discuss, but still they’re able to get the information or get the - educational resources that you’re trying to convey.

MH2

#### Am I Comfortable With the Game Content?

We want to ensure that the content of the game is comfortable to interact with. This includes the content being worded and presented in a way that demonstrates sensitivity. For instance, the game should not betray the user’s trust by suddenly presenting triggering content. Instead, it should allow users to ease into the material:

[Life Goal Cards] could actually cause some people to be anxious, when they’re picking it. And like I could imagine somebody sitting there going `you know, I really wanted to get that degree in computer science, 15 years ago. I never got that degree in computer science.

GD5

#### Am I Comfortable With This Group?

Creating a comfortable social game environment for discussion of sensitive topics is an important and challenging aspect of the game’s design. Initially, we sought information particularly about triggers for individual experiences; however, expert feedback created a better understanding of how the design of the game should be inoculated against situations arising from the social dimensions of a group.

The goal is clear and well-articulated by GD1 in the following quote:

If you can get the group to take it seriously and offer serious conversations, then you can open up things that a group of friends even or family would never talk about normally.

GD1

A challenge could be that people sometimes react in a way that belies our true feelings (eg, laughing because you feel awkward). Moreover, it is possible that a player may react strongly to a general statement (eg, assuming that there is personal commentary, when none exists):

Some people talking about, or sharing, or disclosing—even if it's in an anonymous format—can still have some strong emotional reactions, either to their stuff being selected, or to discovering that that's not the social norm, or to putting themselves out there and not getting the positive feedback that they would hope.

MH3

Moreover, if participants are playing the game in a clinical or formal setting (eg, support group, group therapy, or workshop), where there may be a facilitator or exercise leader, the idea of them not being involved as a player in the game may also be intimidating:

I’d like to have them actually play the game with the participants. That would always be my inclination, rather than kind of standing off to the side observing. Because that obviously leaves a kind of a cold clinical feel to it, like everybody’s - well if they weren’t anxious before, they are now because the psychotherapist is watching. If there was an expert, I’d rather have them play.

MH1

#### Am I Comfortable in This Setting?

After considering the social environment that the game has built for each individual, we moved on to understanding the individual as a group, creating an environment of group comfort.

As MH2 explains, it may be a matter of starting the discussion and allowing it to flourish, suggesting that the game needs only to *spark* the discussion:

It - as far as like stigma, yeah because people get to talk about how they deal with it together. Raise awareness, to have it, normalize it into a game that...yeah. It’s an educational tool. It’s - it - and that, it’s very helpful. It - it makes it so people are talking about it more instead of not dealing with it, so I mean it’s very helpful - it looks like a very helpful tool to use.

MH2

#### Does the Game Need a Facilitator?

A large portion of the discussion arising from the results was the question of whether the game needs a dedicated facilitator.

A facilitator in GD is not an unusual principle. For example, the classic Dungeons and Dragons role-playing tabletop game, has a *dungeon master* role in which one of the players facilitates the gameplay [65]. Commercial games available on the market, such as the Ultimate Werewolf (Ted Alspach, Bézier Games) follows a similar format.

Expert participants provided careful feedback regarding this concept. Points of consideration for the role of the facilitator were related to the maintenance of the comfortable environment:

The only danger might be in - it’s common to any group, where somebody is really insensitive in responding to someone else’s sharings, and they’re like `I can’t believe whoever wrote this was worried about going to the grocery store, like that’s the dumbest thing I ever heard’. That I do wonder - that’s where having a trained group moderator might be helpful.

MH1

In addition to a question of whether the game should be designed with a facilitator role, there was a question about who can fill the facilitator role. Particularly, expert participants cautioned that the situation in which the game is played may require a knowledgeable individual (eg, a clinician or a teacher) to lead the group:

I would also suggest that if this game was being used as anything other than background information for like a health class, then probably it should probably be targeted for use with someone who has competency in delivering it.

MH6

The feedback from the participants about having a facilitator feed into a larger conversation about the overall learning system design. Data demonstrate that there is reasonable concern that a nonfacilitated session depends on the player’s ability to read and interpret information; especially in groups, it is possible that the *loudest voice* may be accepted as correct or misinformation. The game is a learning environment, as such participants should explore the concept and think through the new information; however, participants may not always be trusted to completely read the information available or interpret it correctly.

Moreover, the game presents ambiguous situations with no completely correct answer. For example, the cards may ask about how one would deal with stigma from a family member or in the workplace. In the following quote, GD1 discusses the mechanics of the share card. Each participant provides an answer to the situation, and the game chooses an answer and presents it anonymously:

Sometimes people give advice that comes from a perspective of not really understanding the issues. So I’d be curious if that comes up or how - how the game deals with situations like that. Because there’s no authority of voice here so the game - the game is gonna just pick one at random, right?

GD1

The concern with a game, such as AbW, is that most of the learning occurs within the context of the group conversation and that is encouraged, but discussions cannot be controlled by a card game, even one augmented with an app.

### Learning System

#### Overview

Given that this is an educational game, learning is at the forefront of the game’s goals. We began simply by analyzing the data sets for information regarding the level of content. We then looked at the specificity of the game mechanics to the target group, and finally, we wanted to understand whether the game’s learning system met the learning objectives and if it was replayable.

#### Content and Replayability

The overall goal of the game was to provide information about GAD and PD. Most participants indicated that the content’s depth and breadth were sufficient, with few participants stating that more content should be added and fewer participants indicating that the content was very expansive.

To decide the level of content needed, expert participants pointed to how the game was targeted to a particular subset of individuals:

Having gone through PTSD, someone saying it's an anxiety disorder doesn't teach me anything or feel useful, it just feels a bit dismissive, which seems opposite to your goal.

GD6

The game designer points out in the quote above that there is a difference in how the material feels to someone who is just beginning to understand the problem as opposed to someone who has had lived experience. To a person beginning to learn about clinical anxiety, information about the many subcategories of the large disorder (eg, PD, posttraumatic stress disorder, and GAD) may be surprising.

Participants also mentioned the idea of a facilitator as a way to control the content and flow of the game. That is, if the facilitator is there to control the environment, can they not also control the consumption of information?

Overall, replayability was viewed differently for games designed for general play and nonsensitive topics. By our participants, we are advised that multiple sessions of repeated play can help in effective learning and messaging:

Single run is probably not sufficient or not as good as, but once a week is perhaps too frequent. I would that using the game as part of a process. So, if I meet with a student weekly, that we talk we play the game for some bit. Maybe next week we talk about something else. Maybe we'll explore one of the ideas in greater depth. And then maybe later on, it might be also be group, right. So, it might be individually, and then later it might be some group session and so-on.

MH6

The value of replaying the game for the participants may be understated. Revisiting or replaying the game may help with guidance over a period of growth:

And again with a moderator, a therapist, they might be able to help - actually help that person draw in the positive attributes of their treatment, and how they’re feeling about the treatment they’ve received over a period of time. So whether they can - at that point they would be saying - probably sharing their own experiences, but if - even if they don’t want to share their experiences they would share things that they’ve been told by their therapist, right? So they’ve kind of got to that point where they’re just talking about it, and it might be even helping them destigmatize their own health issues.

GD5

It is possible that as game designers, we see the onus of making the game replayable on the game mechanics, and clinicians see the replayability of the game as an assistant to one’s journey of growth.

#### Target Market, Clinical, and Community Value

From our data, we see that the game, AbW, sits at a branching point in design. Expert participants made us aware that the game could be designed in multiple and highly specific situations. In our results, we captured a large range of use cases. As participants were provided a copy of the game before the interview, many participants came to the interview with ideas for whom the game may be valuable. As our research questions included understanding possible target groups, we probed participants to understand the situations and use cases they had envisioned.

Suggestions included, but were not limited to, therapy groups, support groups, GD workshops for non-MH professionals, in-home family or parent–child dyad communication, and educational institutions such as schools. The diversity of responses was in juxtaposition to the only point of agreement—the game would not be a *pick-up-and-play* game at a friend’s game night or at a game cafe:

As far as the mobile component, like if you can make - maybe if you made the whole game able to be done through an app, people can just meet up for the community - the community focus ones. Like there’s - there’s treatment centres and there’s community focus therapies. So people go to - go to their social work and such and if you have those groups there, you can just pull up the app on their phone and they can all play together.

MH2

When dealing with sensitive topics, our data suggest that the customization of the game should be in accordance with the delicate nuances of the population. These nuances are only revealed when we have gained significant insight into a demographics of participants. These findings are consistent with literature, which indicates that personalization of the learning system is more effective [66].

### Technical and Practical Design

Finally, the last emergent theme that we identified was the technical and practical design. Although, generally, all apps will need some degree of design improvements, here we focus on improvements particular to designing a game on sensitive topics.

#### Onboarding and Instruction

There is a need for intuitive navigation, user interface design, and GD. Here, the focus is on being able to support navigation and research during the game with an emphasis on discretion. This includes being autonomous in leaving and entering the game and not being negatively impacted by accessing resources during gameplay.

Our results indicated that the navigation may be unintuitive for a mixed audience of people with different levels of computer literacy. In the following quote, our expert participant highlights the lack of flexibility in navigation owing to the currently implemented networking structure of the app:

I’m not as technologically sophisticated as the two of you, it takes me forever on my phone to type a message. So if I were to be playing, there would be all these twelve year olds who are done, and I would still be hunting around looking for capitals and punctuation. So that part would be a barrier for somebody like me.

MH3

The goal is to create a pick-up-and-play game to allow participants to concentrate on learning instead of strategy. Again, the contrast between game designers and HPs provided an interesting space for design exploration. Slower and simpler mechanics and pacing were mainly advised by HPs; this was in contrast to game designers, who felt the need to add more mechanisms and allow for divergent strategy and player choice. The divergence among groups of expert professionals converged on the discussions of pacing, emphasizing icebreakers before sharing cards. Allowing to develop a sense of comfort before encouraging more personal discussion was emphasized:

Like once you get started on telling a story - the first time that you tell a personal story should not be to a group of strangers, let’s just - so that’s the risk, right? And so what if the person gets into it and then they get overwhelmed and so they’re getting more and more upset. They’ve said something that’s really upsetting, possibly upsetting other people who have had similar experiences, but even if that doesn’t happen, who’s the person that can handle this and contain it?

MH3

Mediation of these concepts led to the discussion of onboarding or learning to play the game first. Instructions can remove the discomfort of ambiguity:

For the share functionality, the uncertainty here seems like it would be uncomfortable. There aren't clear instructions for who is going to share, or how that should take place. My friends with anxiety all like things to be clear and understandable - uncertainty is a key thing that makes them anxious.

GD7

#### Designing Options

The data revealed that the design of the app should allow more flexibility for players to traverse the content freely during gameplay with other players. The game is designed to link back to resources for further information after the game, but the design should be flexible enough to allow back and forth movement or in-line definitions.

To improve the original design, the game should leverage the moment of curiosity. Acting within the moment to deliver information may be more effective because participants have just uncovered the content and are actively interested:

It’s one thing to have a page of resources but if you can’t access them when you’re not playing the game, then that’s less useful. But if you - if you’re kind of curious is you could have - you know email this to myself or download a PDF onto my phone, then it’s there.

MH3

In the quoted content above, the expert participant makes an important point about note-taking. Participants may want to feel such that they have collected or saved notes during the game.

## Discussion

### Principal Findings

Our results provide us with an insight into the problems that are currently present in the game, AbW; however, the implications of our results are beyond the redesign of a single game. We use AbW as a research probe to understand how to design games for MH and sensitive topics. For each theme, we provide design guidelines for games about sensitive topics. We also provide an example of how to redesign the example game, AbW. Using AbW, we can demonstrate the application of our findings in a redesigning of the game.

In this section, we include a design idea for a possible game for another sensitive topic as an alternative example to demonstrate the applicability of these guidelines outside the game, AbW, showcasing the generalizability of our findings.

### Comfort

By presenting this design guideline, we emphasize that the onus of responsibility to provide this comfortable environment is on the game designers and not the players. Similarly, the designers are also seen as responsible for providing the scaffolding for methods and procedures to maintain a comfortable environment.

#### Focus on the Atmosphere and the Meta-game

Design for comfortable environments that encourage discussion of sensitive topics is a critical challenge to the design of games for sensitive topics. Owing to the sensitive nature of the information, designers need to consider mechanisms for dealing with awkward interactions, icebreakers, and oversharing.

Designing the atmosphere of the game may include considering the design of the system and content of the game and allowing for variables including the group of people, the setting in which the game is being played, and the possibility of a game facilitator.

If we were designing a game that attempts to tackle the topic of racism, it is likely that conversations surrounding slavery, historical violence, and systematic bias in governing bodies would be discussed. For example, given the recent political climate following the brutality of police and murder of George Floyd Jr [67] captured on a cell phone, conversations related to the found footage can be triggering to individuals. Therefore, it is necessary to allow participants to decide the comfortable limitations to the game, possibly by allowing players to create the atmosphere of the game through emergent gameplay.

Consider we propose a design for a board game in which participants are given a large quantity of playable pieces of 8 possible colors. The goal is to fill the board to pursue two possible winning conditions: (1) a single player or alliance of players controls the board and floods the game with a single color, thereby dominating as the majority color until the other players can longer make legal moves or (2) all players win when negotiation leads to an equal ratio of representation of all colors on the board. As participants play token pieces on the board, we may decide to emphasize the injustices faced by minority groups by allowing players represented by the color in majority on the board and allow that player to impose arbitrary overpowered and unfair penalties. As games can become heated, it may be necessary to impose a *clear the board card* or *equal ratios card* to allow the possibility of the second winning condition representing a peaceful ending. Perhaps, a player who is dedicated to pursuing the first winning condition may be *voted out* of the game by a majority vote when the game board is closer to an equal ratio.

A game idea such as this allows players to choose the atmosphere of the game. If the group as a majority prefers collaboration, they may choose to pursue a peaceful ending together as a metaphor for a world with perfect equity.

#### Assign a Facilitator Role to Monitor the Game Environment and Keep a Positive Atmosphere (Optional)

A larger point of discussion within the research was the need for a facilitator. A facilitator is a group leader who would be a helpful factor in maintaining a comfortable environment because responsibility has been assigned.

Expert participants suggested any varieties of facilitation ideas from providing resources to accommodate a player-facilitator to having the game played within a supervised clinical practice. However, convergence on the central need for a facilitator was clear—maintenance and arbitration to maintain a comfortable working environment or the creation of an environment for participants, in which they may feel safe to express their feelings, knowledge, and lived experience. Recommendations from experts in this area suggest the need for a responsible party to act as a group leader—the facilitator.

In the case of AbW, the facilitator role can be added as a nonplayer. The facilitator role allows for the enforcement of a comfortable environment as dictated by the game designer. For example, one option is to limit the game to a clinical setting, played in the presence of a clinician. In this iteration, the information in the rule set on what the facilitator should know, what types of conversations or language to steer away from, or the stipulation that this game should be attended to by a clinician. The clinician, acting as the facilitator, can then use the game as a tool for directed discussion.

Let us propose another GD that may benefit from a facilitator. Perhaps we are hoping to teach players about the diversity that exists in the autism spectrum disorder (ASD). We might decide to extend the known metaphor of a jigsaw puzzle often used by ASD awareness groups by designing a *mystery puzzle*. In the game, participants may be tasked with collaboratively putting together the puzzle without a visual guide. To solve the mystery and piece the puzzle together, participants might be given information from a facilitator. For example, we might have a facilitator who is given special knowledge about the story’s outcome and is tasked with leading other players to the solution by providing hints through telling stories and anecdotes from characters who are identified as being on the ASD spectrum. By promoting empathy through storytelling, the facilitator can complement the collaborative environment needed to piece together a puzzle of an unknown visual outcome.

### Learning System

The core of any educational game is the learning system. In this section, we present the findings that we have concluded from the data to design learning systems within games for sensitive topics.

#### Target the Game to a Player Group in a Specific Setting

In the evaluation of the learning system, we find that there is a need for careful consideration and targeting of the player group because of the sensitive nature of the conversation. Context plays a large role in players’ experience of the game. Playing the game at work, home, school, or a workshop could result in unique experiences with other players and game content. The needs of a single player at these instances change owing to the environment, which encapsulates them. Therefore, player 1 may have a specific set of needs that are divided into player 1.1 in a professional setting, player 1.2 in a social setting, and so on. Hence, there is a need for specific targeting.

Human-centered design, the paradigm of work that is described in the paper by Norman [68] is nearly synonymous with UX, human–computer interaction, and subsequently games user research. However, the targeting recommended by experts in our data set demonstrates that the level at which you specify your user-focus depth and breadth needs to be carefully considered. Comments from experts demonstrate that *a person who is interested in learning* with age-group specifier is not enough. For example, the results suggest that there is a difference between a person approaching this with lived experience and a naïve player with only passing knowledge:

When you get a bunch of people together, and one of those people is a serious anxiety disorder sufferer and everybody else isn’t, then when they talk about their experiences and anxiety, there’s a whole pile of commonality, and one person is thinking `I’m not like these other people, I’m gonna probably keep my mouth shut'.

Participant 3

From the above quote, we see a demonstration of how a group with commonalities in identified target demographics (eg, age) can approach the game and unsuccessfully find common ground.

As game designers, we need to design not only for our target users but also to create an environment in which we can capture a subset of the user’s personality and engage them in a conversation about sensitive topics. Therefore, customization should be based on the common traits of individuals in the target market and not specifically on GD heuristics for that age group.

To apply this design guideline to AbW, we would need to select a targeted setting for the game. For example, redesigning the game to be played with other people in a support group, which meets anonymously at a university space. The game would need to support a larger player group and specifically focus on challenges and emotions, as support groups are designed around discussing the impact of a condition or label and the lived experience of or feelings experienced by the users and design mechanics for player–player interaction. These player–player interactions can be adding a button in the app, which communicates that the feelings or experiences expressed by a player have resonated with others.

As an example of how to apply this principle to other game ideas, let us imagine a game that tackles the topic of children who are terminally ill. We may decide to design the game to be played with healthy siblings or other children visiting the hospital. To do so, we would need to understand the setting of the children’s hospital and the disparity in ability among the groups of players. We may choose to design a game that requires less cognitive effort or physical dexterity, such as games that focus on creative expression and imagination. For example, imagine a game in which participants are using tangram pieces to tell a story. In the game, younger children may have a chance to express their feelings indirectly by telling stories using these large, easy-to-grip, color pieces. Here, this playful activity can be played on small surfaces such as a bedside table or larger surfaces such as the floor.

#### Design Collaborative Challenges

A large body of literature has shown that challenge in games can be important to the motivation to play, the game atmosphere, and player–player interactions [69-71].

A subset of participants felt that the game needed more strategy, in the traditional sense that board games allow participants to occupy their thoughts between their own turns by thinking about their next move. However, AbW’s challenge was the discussion of the sensitive topic of MH:

There’s - this - this to me right now, unless I’m missing something as a naive player, doesn’t feel like there’s any dominant strategy, except for doing exactly the choice that you need to do each round to be able to move forward. So I think from a gameplay perspective, having to make some - some uncertain or unclear choices at some point would enhance the play value, but as often - I’ve seen like with games like this before, I don’t know - I don’t know if that’s requesting too much cognitive lay from people who are already managing a lot of other information, you know. And it also depends on the context and audience. You know, if this is for a discussion group, it is possible that you don’t - you don’t want to put too much game play at the expense of people really just being able to have an experience shared conversation together. So - so that’s gonna shift with your audience, but if it was - if the charge was just increase game play interest level and this, I would have - I would have more choices and more obscured information about what’s the best choice to make was.

GD2

To apply this guideline to AbW, the goal of the game would need to change from *collect all your life goal cards* to *help everyone in the group to collect their life goal cards*. Changing the goal would orient players to a more collaborative environment and complement existing mechanisms for sharing and discussion, such as the share cards or open-hand card game protocol.

We present an alternative example of how this could be used is designing a game to discuss privilege, which can be considered as personal factors, which convey advantage in a situation. As a thought experiment, consider a massive multiplayer web-based game world (eg, Elder Scrolls Online, Bethesda 2014), and imagine instead of the game asking players to choose their race, they were randomly assigned one. Furthermore, let race X be extremely disadvantaged, unable to fight against other races in hand-to-hand combat, and reduce playability of the game. Instead of making the goal *triumph over others in hand-to-hand combat*, consider making the goal *design an arena system with hand-to-hand combat that balances the scales for players who randomly were born into X race*. Changing the goal of the game from individualist goals to collective player goals creates an entirely different game, and this new game creates a better environment for the discussion of privilege by abstracting the conversation from personal traits to in-game traits.

### Technical and Practical Design (Autonomy and Divergent Paths)

The following section refers to the design of interface, navigation, and meta-game environment (eg, how the game is physically situated based on how we expect players to set up the game). In this section, we also discuss practical aspects of researching and designing the game itself for transparency and replicability.

Navigation design is especially important because participants will want to discretely access information during gameplay. We also need to allow for an *escape route* or a method for participants to exit if they are uncomfortable.

For AbW, we may apply this principle in the redesign by allowing participants to change the topic of a share card by anonymously triggering a redraw. To disguise player identity, we may also use the app to randomly call a redraw at times. This would allow participants to avoid conversations based on their own preferences and potentially pass the blame for abrupt change of subject.

We may apply this principle to other game ideas as well. Imagine a game in which web-based players can explore an open world such as a massive multiplayer RPG. Storylines written in the game can tackle sensitive issues through web-based role-play; however, common to massive multiplayer RPGs, there is freedom to choose what areas of the map to explore, form a guild or team with other players, or follow a narrative story path.

### Guidelines for Researching Pregame Design

#### Overview

The review of the literature presented above provides credence to the importance of games user research. In turn, GD should also be based on research. Simply, this may be researching the persona of the target audiences or gathering information for the story based on real-world objects. Researching before designing games only becomes more important as we add purpose outside the entertainment value. As our purposes shift to more serious and sensitive topics, our results demonstrate that games for sensitive topics especially need to be researched.

When researching a sensitive topic with the intention of designing a game, consider the diversity of the topic as it spans multiple points of further inquiry. Results from the 2 expert participant groups allowed for triangulation and insight into the technical and practical design of the game. Therefore, we suggest guidelines for research.

#### Research Diversely

To research diversely means to collect data beyond the scope of information presently captured in one field (eg, GD or health). To implement this principle, the scope of the research must be open to all relevant fields and experts within the scope of the project. Doing so allows one to design a game based on a wide variety of expertise and diversify research beyond general field knowledge and GD.

In other words, game designers should not claim expertise beyond game designing without formal training, hence the need for expert reviews.

To apply this design guideline, we must consider which professionals engage in the topic materials. In the case of AbW, this meant including data from both game designers and MH professionals. It is evident from our results that these 2 groups of experts provided different information that could be contrasting or complimentary at times. To apply this guideline to improve *AbW*, future work may consider interviewing another set of expert professionals: social workers, personal support workers, registered practical nurses, and registered nurses. Unlike other MH professionals interviewed in this study, these individuals have a role in extending care, beyond the individual, to the family as a whole. These experts would be able to provide insight on not only the individual’s health journey but also the individual’s effect on their own community [65].

As an alternate example, consider a game about *sexual orientation and LGBTQ2++ lifestyle*. As usual, game designers will look at the literature, existing state of the art, and other GD professionals; however, we might also ask physicians, psychologists, and neurologists. Moreover, a game designer may also find that activists, women’s and feminist studies scholars, community leaders, and club owners may provide insight into the complex and wide-reaching aspects that encompass the life of an individual belonging to the lesbian, gay, bisexual, transgender, and queer 2++ community.

#### Beware of Assumed Specialization and Misconceptions That Surround the Game When Conducting Research

During the development of AbW and the presented research project, we spoke to many individuals. We consider the assumptions of individuals to whom we communicated our research ideas. During the development and research of the game, AbW, it was apparent that a game for MH was assumed to be for individuals with MH concerns. Ironically, the game was targeted at the exact people who assumed this to battle stigma. Ideally, the game would communicate that it is the community that needs to be informed, work together, and battle misinformation. The target is to impart an understanding that MH is a part of overall health and to restructure the idea that MH is a condition that affects only a subset of people and instead think of MH similar to the way we think of any physical aliment. For example, consider the common cold. At any time, a subset of people might be battling a cold; however, any individual in our community could catch a cold. Therefore, all community members should recognize the basic signs and symptoms of a cold and know basic information about treatment options and where to seek help. Similar to the common cold, a community should also treat individuals with MH concerns as working toward recovery.

During the course of the study, we found that both HPs and game designers got the impression that the game was designed to be a treatment or clinical tool despite our emphasis on it being a game for education. Expert participants were not immune to the assumption, and some expert participants expected the game to be played with a clinical target, even when specified otherwise. To apply this design guideline to AbW, we would see this misclassification as a need for better clarity in the presentation of the game.

As explained in the results, the game, AbW, is at the crossroads of development and therefore one way to counter the assumptions that follow this game (ie, AbW is a game only for people with MH concerns) and apply this design guideline would be to rebrand the game. For example, we may consider a larger group size, a younger audience [13-17], and a school setting. Then, the game would be for general education of high school children during a health or gym course session.

As an alternative example, consider a game being designed for education about a health condition such as type 2 diabetes. The assumption may be that the game would educate individuals newly diagnosed with this condition. However, this disease is likely preventable with lifestyle modifications such as a healthy diet and exercise [72]. Therefore, the game being designed could be for a larger audience. Designers would need to decide, in the early design stages, to determine whether the game will be marketed to all individuals or only those who were recently diagnosed.

### Limitations

Our study combined expertise from multiple disciplines to yield multiple perspectives on the same set of information. As explained in the aforementioned discussion section *(Research Diversely),* increasing the participant sample to a new domain of expert participants will yield a new viewpoint on the same problem. For example, interviewing social workers [73] may yield new information. Future research should aim to gain these additional perspectives.

Moreover, a counter methodology to presenting a cultural probe (as we have done in this study) may reveal additional insights that are not considered by the game designers. For example, the use of participatory design methodology [74] may reveal interesting insights and approaches.

### Conclusions

On the basis of our presented study, we discuss the design of games for sensitive topics. We used the bespoke game, AbW, as a research probe. Our work resulted in the contribution of guidelines that focus on the comfort of participants, optimization of the learning system, and technical and practical guidelines. We made our findings accessible by providing examples for their applications, as applied to AbW, and suggested a possible game idea to germinate creative thinking in our readers.

### Comparison With Previous Work

Previous work on this project was presented as part of a Association Computing Machinery (ACM) special Interest Group (sig) Computer Human Interactions in Play (CHI PLAY) Student Game Design Competition (SGDC) [59] and a conference demonstration with accompanying short paper [17]. These articles can be referred to as earlier versions of the game presented with accompanying documentation and conceptualizations. In this paper, we see the work in its most recent iteration evaluated. Through the process of analysis, we contribute a better understanding of how to research and design games for sensitive topics and MH. In addition, we solidify our conclusions and make them accessible with examples, to iterate on both the presented project and alternative game examples.

## Appendix

Multimedia Appendix 1 Related publications and media.

Multimedia Appendix 2 Interview questions.

## Abbreviations

AbW: Above Water
ASD: autism spectrum disorder
BYOD: bring-your-own-device
GAD: generalized anxiety disorder
GD: game design
HP: health professional
MH: mental health
MHL: mental health literacy
PD: panic disorder
RPG: role-playing game
UX: user experience
VR: virtual reality
