# Supplementary material for: Designing a Serious Game (Above Water) for Stigma Reduction Surrounding Mental Health: Semistructured Interview Study With Expert Participants
Source: JMIR Serious Games. 2022 May 19;10(2):e21376. doi: 10.2196/21376 (PMC9164096; doi:10.2196/21376)
Supplement: Multimedia Appendix 1 [file games_v10i2e21376_app1.docx]

**Multimedia Appendix** **1: Related Publications and Media**

The following appendix contains supplementary materials for the presented paper. Above Water is part of a larger research project, related papers. To request a copy of the game or for more information about the research, please see https://rinawehbe.ca.

**Video Trailer**

Follow this link to watch the video trailer on YouTube: https://youtu.be/oVosNfc6v38 or accessed the archived version of the files in the ACM Digital Library https://doi.org/10.1145/2968120.2971804.

**Related Talks and Publications**

Wehbe participated in a online health seminar Publications on Above Water’s designer interactions [60]: https://doi.org/10.1145/2992154.2996882, https://doi.org/10.1145/2968120.2971804
