# Supplementary material for: Designing a Serious Game (Above Water) for Stigma Reduction Surrounding Mental Health: Semistructured Interview Study With Expert Participants
Source: JMIR Serious Games. 2022 May 19;10(2):e21376. doi: 10.2196/21376 (PMC9164096; doi:10.2196/21376)
Supplement: Multimedia Appendix 2 [file games_v10i2e21376_app2.docx]

**Multimedia Appendix 2: Interview Questions**

The following questions were the basis of the semi-structured interview. As the interviews continued the questions were adaptive to the patterns of themes that developed as the data was collected.

Pre-interview participants were asked to sign a consent form. The interview took place either at the office of the participant or interviewer. Alternatively, if participants wished it, participants could opt for interviews via telecommunication software (e.g. Skype) or written communication (e.g. email).

| - Do you have any questions about the game for me? |
| --- |
| - Do you have any general comments about the game after watching the video and seeing it in action? |
| - Are there specific aspects that you would like to be more in-depth? |
| - Target audience: At its current stage, who do you think would find this game most appealing?  Prompt: Kids / teens / adults / therapy professionals? |
| - If we were to develop this into a game specifically for X audience, what would be the ideal changes? |
| - Is the gameplay engaging? |
| - Can you think of any effective ways to promote this game for the general public to want to actually play it and pick it up? |
| - How did you find the mini-games and the myth-busting segments? Prompt: Are the interesting facts helpful/accurate? |
| - What do you think about each of the cards?  Prompt: Treatment, Anxiety, Share, Life Goals |
| - Do you think enough is covered in this game?  Prompts: Is it too broad/not broad enough? Enough content? Is it lacking any treatment methods that we should be educating people about, or other common things that provoke anxiety, things like that? |
| - Beyond simply educating people, do you think this game model has potential to effectively battle stigma or increase awareness about anxiety? (Prompt: What about using the game to "normalize" anxiety for those already with the disorder  Prompt: help with self-acceptance? |
| - Overall is this game going in the right direction, what would you like to see added/improved/etc.? |
| - Risks of playing this game?  Follow-up: How to eliminate/mitigate those? |
| - Regarding the Resources page with helplines and website links, is this a helpful inclusion in the game design?  How do you think we can better encourage people to access these resources? |
| - Do you think the digital aspect of the game is a good tie-in?  Does it add to or detract from the game?  What additional features/changes would you like to see implemented in the digital component? |
| - The game is meant to encourage anonymous responses so people can explore some of their questions about anxiety in a safe anonymous environment.  Does the game's current model allow for enough privacy? Prompt: Would people be willing to share information when playing with friends/therapy group/etc? |
| - Would this game would be more effective in a single run or periodically? Prompt: For example, maybe once a week? |
